# Genome-Wide Association Study of *Septoria tritici* Blotch Resistance in Ethiopian Durum Wheat Landraces

**DOI:** 10.3389/fpls.2017.01586

**Published:** 2017-09-14

**Authors:** Yosef G. Kidane, Bogale N. Hailemariam, Dejene K. Mengistu, Carlo Fadda, Mario Enrico Pè, Matteo Dell'Acqua

**Affiliations:** ^1^Institute of Life Sciences, Scuola Superiore Sant'Anna Pisa, Italy; ^2^Sirinka Agricultural Research Center Woldia, Ethiopia; ^3^Bioversity International Addis Ababa, Ethiopia; ^4^Department of Dryland Crop and Horticultural Sciences, Mekelle University Mekelle, Ethiopia

**Keywords:** GWAS, *Zymoseptoria tritici*, *Mycosphaerella graminicola*, durum wheat, QTL mapping, landraces

## Abstract

*Septoria tritici* blotch (STB) is a devastating fungal disease affecting durum and bread wheat cultivation worldwide. The identification, development, and employment of resistant wheat genetic material is the key to overcoming costs and limitations of fungicide treatments. The search for resistance sources in untapped genetic material may speed up the deployment of STB genetic resistance in the field. Ethiopian durum wheat landraces represent a valuable source of such diversity. In this study, 318 Ethiopian durum wheat genotypes, for the most part traditional landraces, were phenotyped for resistance to different aspects of STB infection. Phenology, yield and yield component traits were concurrently measured the collection. Here we describe the distribution of STB resistance traits in modern varieties and in landraces, and the relation existing between STB resistance and other agronomic traits. STB resistance sources were found in landraces as well as in modern varieties tested, suggesting the presence of alleles of breeding relevance. The genetic material was genotyped with more than 16 thousand genome-wide polymorphic markers to describe the linkage disequilibrium and genetic structure existing within the panel of genotypes, and a genome-wide association (GWA) study was run to allow the identification of genomic loci involved in STB resistance. High diversity and low genetic structure in the panel allowed high efficiency GWA. The GWA scan detected five major putative QTL for STB resistance, only partially overlapping those already reported in the wheat literature. We report four putative loci for Septoria resistance with no match in previous literature: two highly significant ones on Chr 3A and 5A, and two suggestive ones on Chr 4B and 5B. Markers underlying these QTL explained as much as 10% of the phenotypic variance for disease resistance. We found three cases in which putative QTL for agronomic traits overlapped marker trait association deriving from STB GWA. Our results show that the Ethiopian untapped allelic diversity bears a great value in studying the molecular basis of STB resistance and in breeding for resistance in local and international material.

## Introduction

*Septoria tritici* blotch (STB), caused by the ascomycete fungus *Zymoseptoria tritici* (anamorph *S. tritici* and *Mycosphaerella graminicola*), is among the most devastating foliar diseases of wheat. This disease impacts wheat production in Europe, in the Mediterranean area, in Africa, the Americas, and in Australia (Kosina et al., 2007; Dean et al., 2012; Fones and Gurr, 2015) where, under favorable environmental conditions, can cause relevant yield losses (Eyal, 1999; Duveiller et al., 2007). STB causes premature death of wheat leaves, hampers photosynthesis, and ultimately reduces grain production. Both farming practices and weather patterns influence STB disease severity, as *Z. tritici* requires a moist leaf surface for a successful infection, and spreads throughout the crop canopy via rain splash (Gladders et al., 2001; Pietravalle et al., 2003).

The impact of STB on wheat cultivation may surge with the predicted climate change scenarios. Whilst changes in rainfall and temperature would influence STB spread and severity depending on the agroecology under evaluation (Juroszek and von Tiedemann, 2013), higher atmospheric levels of CO_2_ may boost the development of the disease after acclimation (Váry et al., 2015). Concurrently, means of STB control in the field are becoming less sustainable. Fungicides may fail in controlling STB due to the repeated emergence of resistance alleles in the pathogen (Cools and Fraaije, 2013; Torriani et al., 2015). Although some fungicides remain effective, they retain high monetary and environmental costs (Fones and Gurr, 2015). The investment required to control STB by these means is outside the reach of developing countries, where STB severely impacts the economy and food security (Kosina et al., 2007). For all these reasons, breeding for host plant resistance is an appealing perspective to achieve an economical, durable, and environmentally friendly control of STB in wheat fields (Orton et al., 2011; Fones and Gurr, 2015).

Durum wheat (*Triticum turgidum* sub. *durum* Desf.) and bread wheat (*Triticum aestivum* L.) are both hosts of *Z. tritici*, but the latter especially has been used to explore the host–fungus interactions in STB. The inheritance of the resistance to STB is complex and challenging to track (Rosielle and Brown, 1979) also because STB rapidly changes its host-specificity (Stukenbrock et al., 2007), leading to overcoming host disease resistance (Rudd, 2015). Developing a durable resistance to STB is made more difficult by the high levels of genetic variability in *Z. tritici* populations, contributed by frequent sexual recombination (Zhan et al., 2003). So far, 21 genes were identified to confer resistance to STB and were designated *Stb1* to *Stb18, StbSm3, StbWW*, and *TmStb1* (Brown et al., 2015). By now, tens of quantitative trait loci (QTL) for STB resistance have been detected in a number of mapping populations, reporting an extraordinary diversity, and complexity of the genetic basis of STB resistance (Brown et al., 2015). Ever more, there is the need to put to use this information to produce resistant wheat genotypes. The recent availability of high-definition genotyping platforms (Wang Q. et al., 2014) discloses new perspectives for the screening of untapped genetic material and for the high-definition identification of genomic loci responsible for STB resistance. Dense markers were used in genome-wide association (GWA) studies to detect loci involved in STB resistance on diversity panels (Kollers et al., 2013; Gurung et al., 2014; Arraiano and Brown, 2016). These methods provide the means to speed up the production of resistant varieties through marker-assisted selection (MAS) or other biotechnological approaches. Single nucleotide polymorphism (SNP) markers identified in GWA studies can be used to produce efficient tools for MAS in breeding, such as competitive allele specific PCR (KASP) markers. KASP markers developed from SNP sequences flanking the putative QTL regions allow MAS to efficiently track the inheritance of the desired traits, being them agronomic (Cabral et al., 2014) or disease related (Dreisigacker et al., 2015; Chhetri et al., 2017).

Ultimately, the improvement of wheat cultivars for STB resistance is dependent on the availability of resistance traits in the breeding material considered. Wheat landraces are a good source of resistance alleles for fungal pathogens (Ghavami et al., 2011; Cavanagh et al., 2013), and STB resistance exists in local durum wheat cultivars from Iran (Ghaneie et al., 2012) and Tunisia (Ferjaoui et al., 2015). We recently characterized the genetic diversity of a representative collection of Ethiopian durum wheat landraces, reporting their uniqueness in relation to international durum wheat germplasm (Mengistu et al., 2016). Further studies confirmed Ethiopian wheat diversity in relation to international material (Kabbaj et al., 2017). Ethiopia is a center of diversity for tetraploid wheat (Vavilov, 1992), and so is for wheat pathogens (O'Donnell et al., 2008; Kolmer and Acevedo, 2016; Wan et al., 2016). Local durum wheat landraces have been grown and selected under combined natural and anthropic pressures in a low-input agriculture system, developing multiple resistances to local pathogen strains, including the devastating Ug99 stem rust disease (Klindworth et al., 2007).

The aim of this study was to exploit the phenotypic and molecular diversity of untapped Ethiopian durum wheat germplasm to identify genetic loci relevant to STB resistance. To do so, three STB resistance traits were scored in a panel of 293 landraces and 25 modern varieties under natural infestation for 2 consecutive years, in an open field location in the Ethiopian highlands. Several landraces appeared resistant to STB. Agronomic traits were simultaneously measured on the same panel, allowing to study the relation between resistance traits and agronomic traits in the Ethiopian durum wheat landraces material. Using extensive genome-wide molecular markers, a GWA scan was conducted identifying five genomic loci associated with the severity and progression rate of STB disease, two of which not yet reported in the literature. Additionally, we discuss suggestive resistance loci. These resistance loci may be relevant for breeding pipelines aiming to improve the resilience of Ethiopian and global durum wheat material to this devastating disease.

## Materials and methods

### Plant materials and genotyping

The diversity panel employed in this study comprises 293 Ethiopian durum wheat landraces and 25 Ethiopian durum wheat improved varieties (Table S1). The panel was assembled to represent Ethiopian durum wheat diversity. Landraces were obtained from the Ethiopian Biodiversity Institute (www.ebi.gov.et) in form of seeds. During the prior seed amplification, each accession was cleaned to obtain a reference genotype that was used for genomic DNA extraction and for field experiments downstream. Full details about the diversity panel assembly procedure can be found in Mengistu et al. (2016). Genomic DNA was extracted using the GenElute Plant Genomic DNA Miniprep Kit (Sigma-Aldrich, St Louis, MO) in Mekelle University, Mekelle, Tigray region, Ethiopia. After the check for quality and quantity, the DNA was typed with the 90K SNP wheat array (Wang S. et al., 2014) at TraitGenetics GmbH (Gatersleben, Germany). Samples not providing genotypic data of sufficient quality were discarded from the molecular analyses. SNPs were filtered for failure rate below 20% and for minor allele frequency above 5%. Details about the genotyping procedure can be found in Mengistu et al. (2016). Complete genotyping data for the varieties included in this study is reported in Table S9.

### Field experiments

The experiment was conducted under natural infestation during 2012 and 2013 growing seasons at Geregera, Meket district (Wollo, Amhara region, 11°4′N/38°52′E; WGS 84 coordinates), at an altitude of 2,876 m above sea level. The experiment was conducted in rainfed conditions. Sowing was performed after the onset of the main rainy season, on July 5, 2012, and July 9, 2013, respectively. The field was laid out in a partially balanced lattice design, with replications of block size 20 rows by 20 columns, block length 49.5 m. The genotypes sown but not used for this study were considered as fillers. This design was used to allow the adjustment of treatment means for block effect as well as to provide effective control within replicate variability. The seed rate used was 85 kg ha^−1^, with seeds drilled evenly in rows. Recommended rate of fertilizer for wheat for Geregera area were used, with 64 kg N (Urea) and 46 kg P_2_O_5_ (DAP) per hectare. P_2_O_5_ was entirely applied at planting time, while N was applied 1/3 at planting and 2/3 at tiller initiation. Weeding was done three times by hand. In 2012, the plot size was 2 × 0.6 m, and each plot had three rows with 0.2 m spacing. In 2013, the plot size used was 2 × 0.8 m, and each plot had four rows with 0.2 m spacing. The spacing between rows and replications were 0.5 and 1.5 m, respectively. Five plants in the middle rows were randomly selected and tagged to be used consistently to record STB infection. The same field was used to collect agronomic and phenologic traits.

### Evaluation of STB infection

The Septoria disease severity (SDS) was scored visually, according to a double-digit scale (00–99) modified from Saari and Prescott (1975) for wheat foliar diseases. The assessment was taken in five randomly selected productive plants. The evaluation was carried out individually at two time points: the heading stage and the maturity stage. For each score, the percentage of disease severity was estimated based on Equation (1), following the formula used by Sharma and Duveiller (2007):

(1) $$SDS=\left[\left(\frac{D1}{9}\right)\left(\frac{D2}{9}\right)\right]100$$

where *D1* represents the vertical disease progress as deriving from the average relative height reached by the disease as recorded from five random plants (0–9). *D2* represents the severity of the disease, measured as the average relative coverage of the diseased leaf area (i.e., the necrotic leaf area) recorded from the upper four alive leaves in the same five plants. The SDS index is thus composed by a first digit representing the blotch development up the plant height (e.g., 5 if the disease reached the mid-point of the plant or 50%, 8 if it reached the flag leaf, 9 if it reached the spike), and a second digit representing severity *per se* (e.g., 1 for 10% to 9 for 90%). SDS values range from 0 to 100, where 0 would indicate complete resistance, and 100 would indicate complete susceptibility. The SDS trait was sided by the Septoria progress coefficient (SPC) trait, calculated as in Equation (2) following the formula from Eyal and Ziv (1974):

(2) $$SPC=\left(\frac{SDH}{PH}\right)$$

where *SDH* (Septoria disease height) is the maximum height from the ground where pycnidia of the pathogen are found on the plant, in cm. *PH* is the height of the accessions, averaged over the same plants used to derive *SDH*. The coefficient indicates the position of pycnidia relative to plant height, regardless of pycnidial coverage, and allows the comparison of infection placement on cultivars with different plant stature. SPC is thus a ratio indicating the height reached by the disease. SPC of 0.0 would mean no disease at all, while SPC of 1.0 would indicate that the disease is covering the entire plant.

Ten traits accounting for phenology, yield, and yield components were investigated. Days to flowering and days to maturity were recorded for whole plots when 50% of the plants reached the corresponding Zadock's growth stage. The full plot was used to measure grain yield (as grams of grain produced per plot, then projected to t/ha), biomass (as the dry weight of the above-ground harvested biomass per plot, then projected to t/ha) and 1,000 grain weight (as the weight of 1,000 kernels, in grams). The moisture content of grains was measured using a digital grain moisture meter and used to adjust grain yield and biomass to 12.5% moisture content. Three randomly selected plants per plot were used to measure tillering (as the number of effective/productive tillers per plant), plant height (in cm), spike length (distance between the pedicule base and the tip of the spike excluding awns, in cm), number seeds per spike, and the number of spikelets per spike. For further details about the agronomic data collection, see Mengistu et al. (2016).

### Phenotypic data analysis

All phenotypic data was normalized using arcsine square root transformation. The phenotypes were analyzed using a linear mixed model (LMM) including genotypes as fixed effects. The effects of year, block and the interaction of genotypes by year were treated as random effects. The parameters of the model were estimated by the method of restricted maximum likelihood (REML) using SAS statistical software (SAS Institute, Cary, NC). BLUE values were calculated from replicates of each line for each trait for the different trials. Test of *X*^2^ goodness of fit was applied to investigate if there was normality performance of each trait at each trial. BLUE values are referred to as “combined phenotypes” throughout the manuscript text. Phenotype means were transformed back to percentages for discussion. The software R (R Core Team, 2013) was used to conduct the analyses and produce plots with custom script available upon request. Histograms were plotted using R/ggplot2 (Wickham, 2009), correlations were plotted using R/corrplot (Wei, 2013).

### Genome-wide association study

Association mapping was conducted using R/GAPIT (Lipka et al., 2012). The initial 81,584 markers available on the array have been filtered to produce the working SNP set used in downstream analyses. Markers failing in over 20% of the samples were removed. Markers having a minor allele frequency below 5% were also filtered. Map position of markers was derived from the consensus durum wheat genetic map (Maccaferri et al., 2015). Pairwise LD measures (*r*^2^) were obtained for all markers in each linkage group using R/LDheatmap (Shin et al., 2006). To calculate LD decay, only markers pairs within 50 cM were considered. Mean LD for pairs was calculated for markers at increasing genetic distances with a sliding window and plotted against the genetic distance with custom R scripts available upon request.

The working set of markers was input together with STB resistance phenotypes combined over the 2 years into a mixed linear model (MLM) accounting for uneven relatedness among samples. This method effectively controls population structure to lower type I errors. Kinship was calculated in R/GAPIT following VanRaden's method (VanRaden, 2008). Principal components (PC) describing the genetic structure of the panel were calculated with R/GAPIT and iteratively added to the fixed part of the model, from PC1 to PC10. The SUPER compression model was used Wang Q. et al. (2014). The best fit of the model was visually evaluated on quantile–quantile plots. Marker-trait associations (MTA) were deemed highly significant when surpassing a threshold calculated using the Bonferroni method and accounting for multiple testing at a nominal *p*-value of 0.15. GWA scan providing significant MTAs according to this threshold are the sole reported. The false discovery rate (FDR) was computed using Storey's method (Bass et al., 2015) and was reported with GWA results as an alternative, less stringent multiple testing correction method. In order to provide a better description of the genomic locations associated with SDS and SPC, the same GWA model was run on data collected each year separately and on agronomic trait values combined over the 2 years. Allelic states at significant markers were converted to the arbitrary numeric values (−1, homozygous for the most frequent allele; 0, heterozygous; +1, homozygous for the less frequent allele). *R*^2^ values for significant marker tests were produced regressing numeric allele states at the markers over the corresponding phenotypes with a linear model. The regressions coefficient was then used to derive positive alleles at each significant MTA.

Significant markers without map positions were traced back on their genetic positions by using their numerical encoding as phenotypes in a GWA scan using a compressed linear model in R/GAPIT. The deriving MTA denote genomic regions in LD with the unmapped marker used as phenotype, and thus indicate the likely genetic position of the latter. Custom R scripts available upon request were used. A putative QTL is defined as a genomic location identified by one or more MTAs.

KASP markers were designed from SNP markers underlying putative QTL for STB. When possible, multiple markers per QTL were developed. KASP markers were designed and tested on a set of 46 Ethiopian samples belonging to the durum wheat reference collection (DWRC) at LGC (http://www.lgcgroup.com/). Working KASP markers are reported in Table S8.

## Results

### Phenotypic data analysis

**Figure 1:**
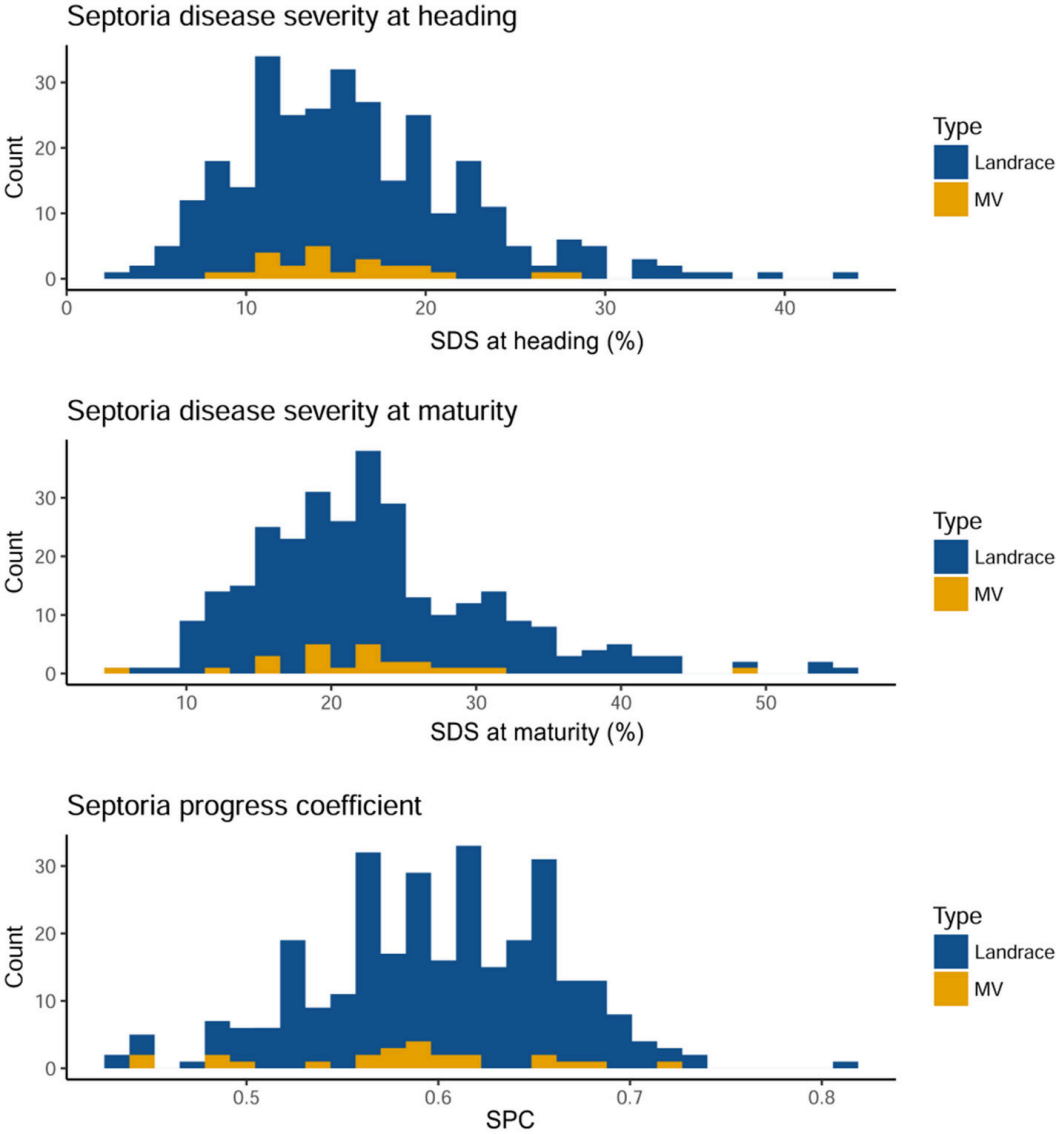
Distribution of the disease traits combined over two consecutive years on landraces and modern varieties (MV). Higher affection classes are found toward the right end of the graphs. Septoria disease severity at heading and maturity follow a normal distribution. At maturity, the severity distribution is expectedly shifted to higher values. Septoria progress coefficient, a measure of disease severity normalized by plant height show a different distribution. On the x-axis, the BLUP value of the considered genotype. Colors according to legend.

*S. tritici* blotch (STB) natural infestation was slightly different over the 2 years. An ANOVA showed that the genotype effect was significant in each year, and that the year effect was significant when considering combined data, except for SDS at maturity (Table 1). The interaction of year by genotype was significant in all the disease traits analyzed. The distribution of Septoria disease resistance was similar among landraces and MVs for the three traits considered (Figure 1). The distribution of the traits is pseudo-normal, confirming the quantitative nature of Septoria resistance in the Ethiopian material. SDS was expectedly higher at maturity than at heading stage. Ethiopian durum wheat landraces are extremely diverse in their resistance to STB (Table S1). SDS at heading ranges from 3.38 to 43.97% in landraces, whilst in MVs range from 8.45 to 28.22%. A hundred and thirty-five landraces (46.1%) and 13 MVs (52%) show a disease severity below 15% at heading. At this stage, however, only two MV show SDS below 10%, while 51 landraces do. At maturity, the range for SDS ranges from 7.64 to 55.95% in landraces and from 5.65 to 48.96% in MVs. Among landraces, 135 (40.3%) fall within 20% SDS, while five MVs (50%) do. Only one MV but 43 landraces showed SDS at maturity below 15%. The range of SPC is broader for landraces (0.43–0.81) than for MVs (0.45–0.72). In 2012, the MV with the lower SDS at heading was *Mangudo* (8.45), while the MV with lower SDS at maturity was *Selam* (5.65) (Table S1). *Selam* was the MV having the lower SDS at heading and maturity in 2013, and together with *Mangudo* was the most resistance MV across the 2 years' data in disease severity at maturity and heading, respectively. *Tossa* was the most susceptible line for SDS at both heading and maturity across the 2 years. In many cases, the same varieties had different affection severities across the 2 subsequent years. This can be seen comparing SDS and SPC values for the same genotype across the two seasons (Figure S1). Although the pressure of disease at heading and maturity was generally lower in 2013 than in 2012, several varieties showed contrasting trends resulting in higher SDS values in 2013 than in 2012, especially at the maturation stage. Conversely, SPC shown a generalized increase in 2013 as compared to 2012, with most of the varieties showing increased affection values over the 2 years (Figure S1).

**Table 1:** Partitioning of variance sources of fixed effects and their level of significance for Septoria disease severity (SDS) and progress coefficient (SPC).

	**Variance source**	**d.f**.	**Variance**		
			**SDS at heading**	**SDS at maturity**	**SPC**
2012	Genotypes	318	756.4^***^	923.8^***^	526.7^***^
	S.E.		0.08	0.09	0.07
2013	Genotypes	318	524.6^***^	466.9^***^	605.2^***^
	S.E.		0.09	0.09	0.06
Combined	Year	1	13.4^**^	0.03	34.3^***^
	Genotypes	317	786.9^***^	889.0^***^	679.0^***^
	Genotypes X year	317	469.1^***^	504.9^***^	433.6^***^
	S.E.		0.06	0.06	0.04

*** p < 0.001;

** p < 0.01;

^*^ p < 0.1; S.E., standard error; SDS, Septoria disease severity; SPC, Septoria progress coefficient.

**Figure 2:**
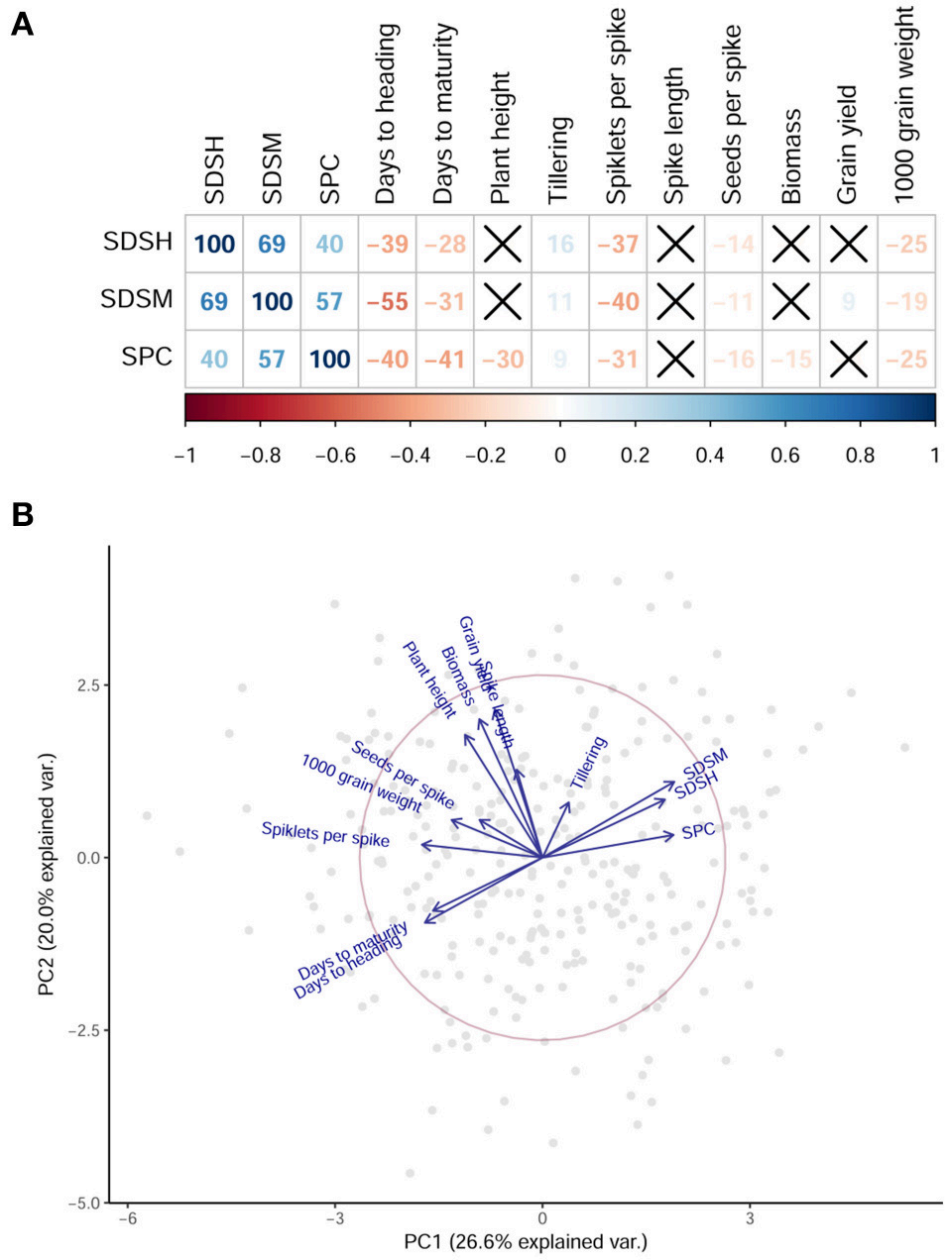
Relationship among disease resistance traits and agronomic traits. **(A)** The results of a correlation analysis on disease traits and agronomic traits. For each pair of phenotypes, correlation coefficients are reported as percentages, and are colored with a shade representing the intensity of the correlation according to the color bar below. Crossed-out squares represent non-significant correlations. SDSH, Septoria disease severity at maturity; SDSM, Septoria disease severity at maturity; SPC, Septoria progress coefficient. **(B)** A biplot of the first two PC representing the phenotypic variance. Gray dots are samples. Blue arrows represent the phenotypes included in the PCA. Early flowering samples have lower disease affection.

Disease resistance traits were correlating with some of the agronomic traits measured in the same experimental field (Table S2, Figure 2A). The phenology of the plant is linked with the progression of the disease, as reported by a significant negative correlation between SDS and days to flowering and days to maturity. Grain yield and biomass, conversely, show low correlations with all disease traits, and spike length is not significantly correlated to any of those. This result shows that in our panel those genotypes with the highest productivity also suffer the highest STB affection. The number of seeds per spike and the grain size (reported as thousand grain weight) are inversely correlated with SDS (Table S2). A principal component analysis (PCA) was used to visualize the relation between disease and agronomic traits in the collection (Figure 2B). The first two PCs cumulatively explained 46.6% of the total variance. Genotypes having a shorter flowering time also have a higher infection, although the causal direction of this relationship cannot be derived from the data. This could be contributed by disease escape mechanisms, i.e., late flowering varieties escaping the disease spread and appearing more resistant as a consequence (Arraiano et al., 2009). Measures of yield and yield components are only poorly correlated to SPC and SDS.

### Identification of marker-trait associations

**Figure 3:**
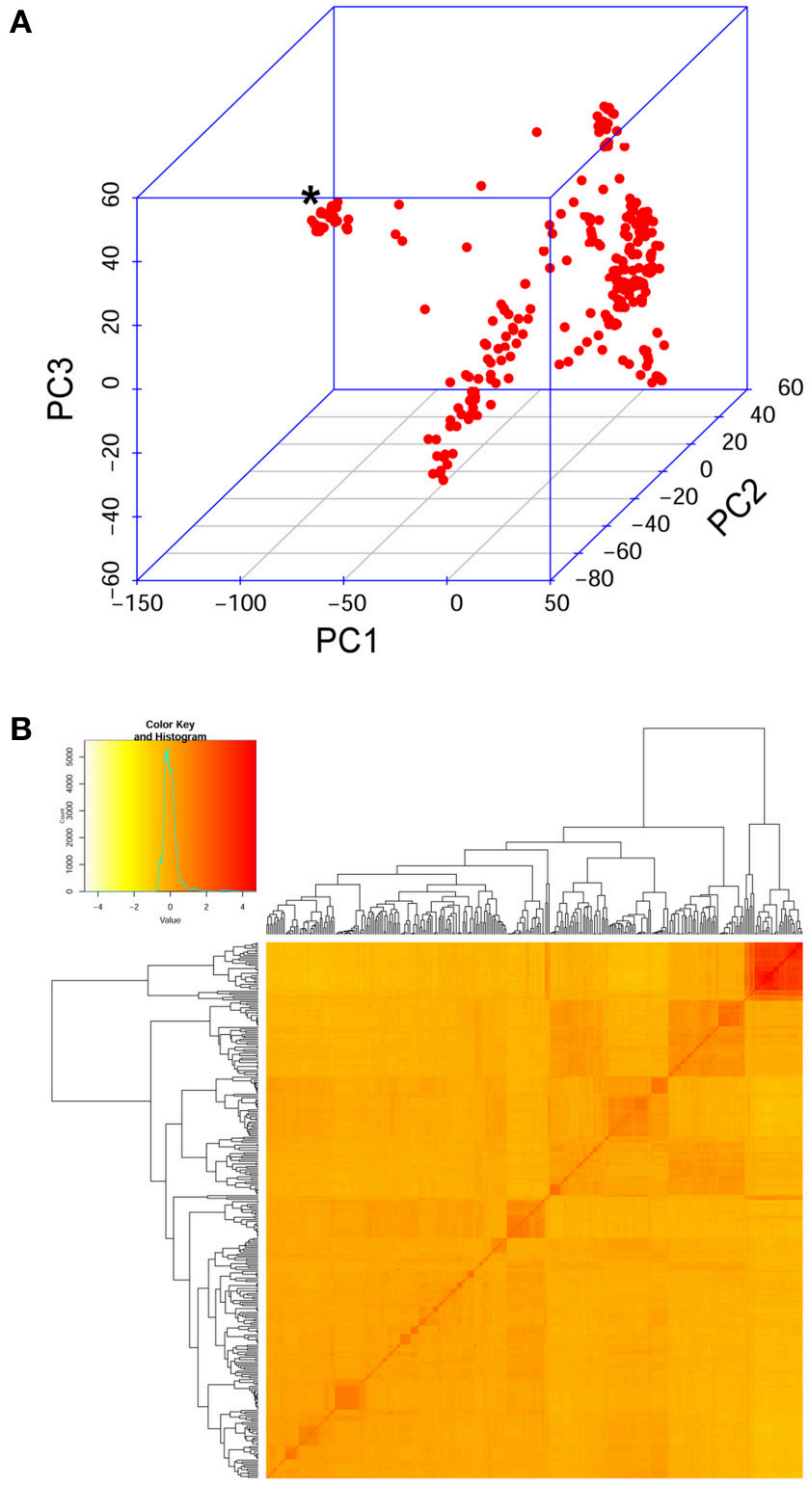
Genetic relatedness in the diversity panel evaluated on the SNP data also used for GWA. **(A)** Samples' relationship in the three-dimensional space of the first three principal components derived from a PCA. Red dots represent samples. An asterisk marks the group of modern varieties, which lay separated from Ethiopian landraces. **(B)** Kinship analysis on the panel. Top left, the distribution of estimated kinship values follows a normal distribution (turquoise curve). In the main panel, the pairwise kinship values depicted in increasing tones of red. Outside the matrix, the resulting clustering tree.

The Ethiopian durum wheat panel was genotyped with the wheat 90K array (Wang S. et al., 2014). After filtering for sample quality, 300 accession were retained for the diversity analysis and the GWA. After filtering for data quality, 16,223 polymorphic SNP markers were retained and used to evaluate the genetic structure of the panel and to conduct a GWA study on the measured phenotypes. The PCA and kinship analysis conducted on molecular data showed that the durum wheat cultivated in Ethiopia is clearly divided into two groups. The group of MVs is highly similar within itself and different from that of landraces (Figure 3A). Landraces, on the other hand, are highly admixed and uniformly differentiated from MVs (Figure 3B). If not controlled for, this structure could inflate GWA statistics.

**Figure 4:**
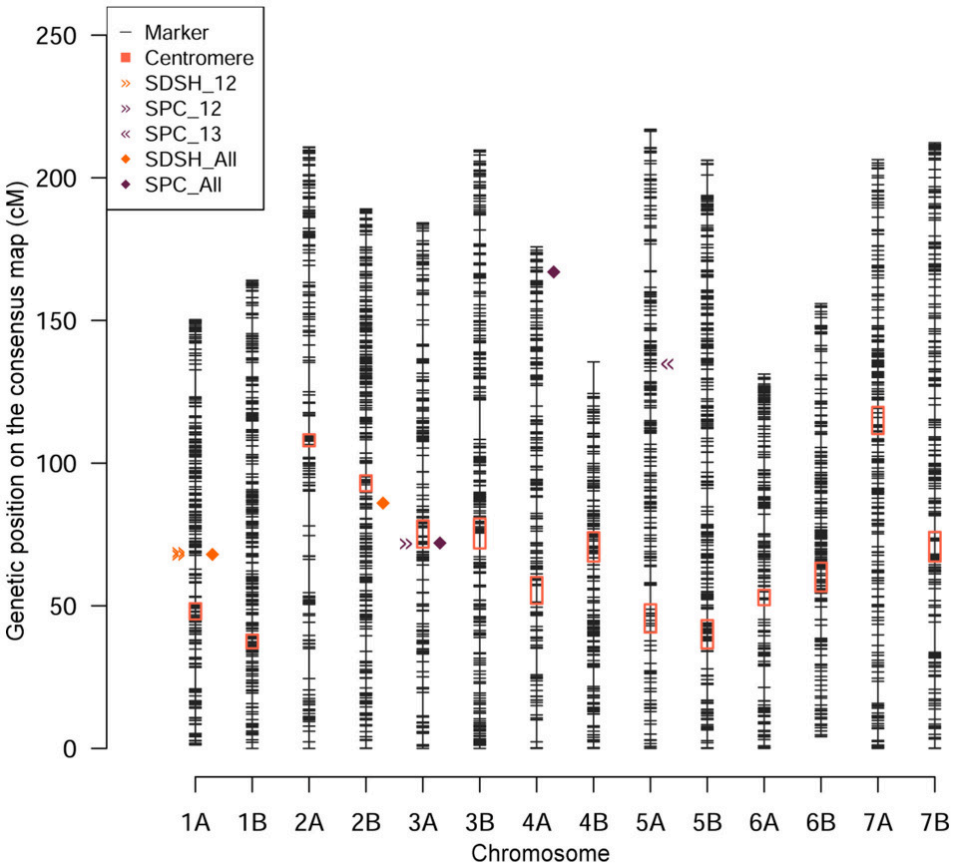
Genomic position of the putative QTL identified for disease resistance. The SNP markers employed are depicted according to their genetic map position (y-axis). Pericentromeric regions are highlighted by red boxes. The genetic position of the putative QTL identified by MTAs are depicted by shapes and colors according to the legend top left.

The GWA was conducted on STB resistance data combined over the 2 years, then on STB data collected each year separately. The GWA scan was performed also on agronomic data combined over the 2 years. We report and discuss marker-trait associations (MTA) surpassing the Bonferroni threshold. Individual marker *p*-values and FDR corrected q values for GWA scan on STB are reported in Table S3. Quantile-quantile plots for selected models are reported in Figure S2 for STB resistance data and in Figure S3 for agronomic data. Overall, 35 MTAs were identified with 32 unique SNP markers, of which 13 did not have a chromosomal position on the durum wheat genetic map (Maccaferri et al., 2015). The position of unmapped markers providing significant associations was derived exploiting their linkage disequilibrium (LD) with markers having a map position: for disease data, they clustered with MTAs already reported on chromosome (Chr) 1A and Chr 3A. A previously unmapped MTA for SDS at heading recorded in 2012 was mapped to Chr 5B (Table S4). The *R*^2^ for MTAs varied substantially among traits and markers, the highest being 10.6% for an SPC MTA on Chr 3A. SDS at heading was the trait providing the second highest *R*^2^, at 8.7% for and MTA on Chr 2B (Table S5). The MTAs were combined according to their genetic position to identify putative QTL for STB resistance. In order to identify a window of genetic distance in which to combine MTAs, a pairwise LD analysis was conducted using the SNP markers produced on the panel. The LD in the Ethiopian durum wheat was dropping below *r*^2^ = 0.2 (representing null LD) within 1.5–6 cM in all chromosomes (Table S6, Figure S4), denoting a relatively high mapping definition. MTAs falling on the same linkage group within the genetic distance for LD decay specific for that chromosome were assigned to the same putative QTL, giving a total of five putative QTL identified (Table 2, Figure 4). A putative QTL deriving from the MTA with estimated genetic position on Chr 5B is discussed separately. Diagnostic KASP markers for qSTB.1, qSTB.3, qSTB.4, qSTB.5, and for the putative QTL on Chr 5B are reported in Table S8.

**Table 2:** Putative QTL identified for STB resistance.

**Putative QTL**	**Map position**	**Number of MTAs**	**Flanking markers**	**Chr**	**Phenotypes**	**Year data**
qSTB.1	67.7–69.2	1 (6)	IACX3496 / Kukri_c10239_2186	1A	SDS at heading	Combined (2012)
qSTB.2	85.8	1	Jagger_c9472_305	2B	SDS at heading	Combined
qSTB.3	71.6–72.5	9 (1)	wsnp_Ex_c3478_6369892/ wsnp_RFL_Contig4404_5157920	3A	SPC	Combined (2012)
qSTB.4	167.5	1	Excalibur_c113341_139	4A	SPC	Combined
qSTB.5	135.2	1	Tdurum_contig10210_425	5A	SPC	2013

*The name, map position (in cM), the number of MTAs, flanking markers, chromosome (Chr), measured phenotype, and year of the data originating the putative QTL are given for each entry. In case the putative QTL derives from multiple datasets, the number of MTAs and the year data for the alternative dataset is given in brackets. SDS, Septoria disease severity; SPC, Septoria progress coefficient*.

Combined measures for SDS at heading identified one MTA pointing to a putative QTL at 67.7–69.2 cM on Chr 1A (qSTB.1) and one MTA at 85.8 cM on Chr 2B (qSTB.2) (Table 2, Figure 4, Figure S5). One additional unmapped MTA for SDS at heading was mapped to qSTB.1 (Table S4). The combined measures of SDS at maturity did not provide any MTA at the stringent significant threshold utilized. However, the manhattan plot for SDS at maturity clearly show a significance peak barely missing the threshold at ~4 cM on Chr 4B (Table S3, Figure S5). Although not significant in absolute terms, the shape of this this peak is suggestive of the presence of multiple markers in LD with a genetic element influencing the phenotype. The combined measure of SPC, representing the relative height of the plant reached by the disease, identified two major putative QTL at 71.6–72.5 cM on Chr 3A (qSTB.3) and at 167.5 cM on Chr 4A (qSTB.4), supported by nine and one MTAs, respectively (Table 2, Figure 4, Figure S5). Combined SPC identified also 11 unmapped MTAs that were traced to qSTB.3 (Table S4).

In order to provide a finer dissection of the molecular basis of STB resistance in Ethiopian durum wheat landraces, the GWA scan was repeated on STB data specific to 2012 (Figure S6) and 2013 (Figure S7). Although single-year GWA data allows a lesser degree of generalization, it may account for yearly specificity in STB infections. The SDS at heading in 2012 also identified qSTB.1, reported by the combined measures over the 2 years as well (Table 2, Figure 4). In this case, the putative QTL was supported by six MTAs. SDS at heading in 2012 identified one MTA without genetic position that we mapped at 43.4 cM on Chr 5B (Table S4). The same phenotype measured in 2013 did not provide associations significant at the Bonferroni threshold. SDS measured at maturity in 2012 and 2013 and independently used in a GWA scan did not identify any significant MTAs. The SPC measured in 2012 provided a strong MTA supporting qSTB.3, also identified by the combined measures of SPC (Table 2, Figure 4). The MTAs without map positions also resulted overlapping qSTB.3 (Table S4). SPC data collected in 2013 identified one putative QTL at 135.2 cM on Chr 5A (qSTB.5) (Table 2, Figure 4).

Significant associations deriving from agronomic traits reported *R*^2^ as high as 26.6% in the case of days to maturity, and above 26% in the case of several markers associated to 1,000 grain weight and number of spikelets per spike (Table S5). Days to heading provided three strong signals on Chr 1B at 81 cM, Chr 2B at 80 cM, and Chr 4B between 3 and 6 cM (Table S7, Figure S8). Days to maturity identified two putative QTL on Chr 1B at 35 cM and on Chr 4A at 51 cM, respectively. A highly significant putative QTL was identified from the GWA scan for plant height on Chr 3B at 166 cM. Spike traits are contributed by several major putative QTL (Table S7, Figure S9). Seeds per spike identified significant MTAs on Chr 3A at 141 cM, on Chr 4A at 170 cM, Chr 4B at 52 and 87 cM, Chr 5A at 14 cM and 142 cM, and on Chr 5B at 43 cM. Spike length identified a unique MTA on Chr 4B at 91 cM. Four putative QTL were identified by measures of 1,000 grain weight on Chr 1A between 60 and 70 cM, on Chr 5A at 44 cM, on Chr 5B between 38 and 41 cM, and on Chr 6A at 85 cM. The number of spikelets per spike provided suggestive peaks on Chr 1A and 4A, but no highly significant putative QTL.

In few cases, the putative QTL provided by disease data overlap those deriving from agronomic measures. The putative QTL for seeds per spike on Chr 4A is proximate to qSTB.4, identified by combined measures of SPC (Table 2), and that on Chr 5B co-maps with the previously unmapped MTA for SDS at heading in 2012 (Table S4). The putative QTL mapped on Chr 1A for 1,000 grain weight co-maps with qSTB.1, and that on Chr 5B overlaps that for seeds per spike and the previously unmapped MTA for SDS at heading in 2012 (Table S4). Putative QTL from phenology and plant height do not co-map with any STB MTA.

Several unmapped MTAs for agronomic data were traced to their most plausible genetic position, many of which overlapped putative QTL identified by mapped markers (Table S4). Novel genomic loci of agronomic significance reported by MTAs with estimated map positions include several putative QTL for seeds per spike, at ~108 cM on Chr 2A, 162 cM on Chr 4A, 0, 18 and 60 cM on Chr 4B, and 95 cM on Chr 7A. An MTA mapping at ~40 cM on Chr 4B is jointly identified by 1,000 grain weight and seeds per spike.

## Discussion

Here, the diversity of Ethiopian durum wheat was described in relation to open field STB infection. The experiment, conducted for two consecutive years on the Ethiopian highlands, is the first exploring the genetic bases of STB resistance in Ethiopian material representative of the diversity of durum wheat cultivated in the region. Considering the fact that we previously demonstrated how Ethiopian durum wheat gene pool is genetically distant from that commonly employed in breeding efforts (Mengistu et al., 2016), and considering the high-density molecular characterization employed for the first time in a GWA study on STB resistance, we expected to identify new and different putative QTL to that reported in literature. Although the population structure existing in the panel was accounted for (Figure 3), the quantile–quantile plots deriving from the GWA analysis still showed some inflation (Figures S2, S3). In order to correct for this inflation, and to reduce Type I errors, a conservative Bonferroni threshold was employed (Johnson et al., 2010). Upon visual evaluation of the most suggestive peaks reported by the manhattan plots resulting from the GWA (Figures S5–S7), the nominal *p*-value to be corrected for multiple testing was set to be 0.15. This allowed us to mitigate Type II errors and to report outstanding MTA without including background noise. In order to further reduce the incidence of false negatives, we report an FDR correction for multiple testing (Bass et al., 2015) for each marker test in Tables S4, S5. The use of the Bonferroni threshold resulted in 35 MTA detected for different aspects of STB resistance. Those having a position on the durum wheat genetic map provided altogether five highly significant putative QTL (Table 2). Those without a map position on the durum wheat genome were traced back to qSTB.1 and qSTB.3, and additionally at ~40 cM on Chr 5B.

The fact that each of these QTL maps on a different chromosome and that the *R*^2^ of associations for disease traits are mostly below 10% (Table S5) confirm the quantitative pattern of inheritance for STB resistance (Brown et al., 2015). This is possibly contributed by the existence of different resistance genes responding to different Septoria natural isolates, whose effect cannot be untangled in open field infestation. Though several major resistance genes for STB have been described in hexaploid wheat, it is the first time that QTL related to STB resistance are reported in Ethiopian durum wheat. The diverse types of marker, mapping methods and populations used in wheat genetics make it difficult to determine whether QTL identified at approximately in the same position in different studies are indeed the same, even if comparative maps are available (Maccaferri et al., 2015). The comparability of our results to previous literature on STB resistance is also made more difficult by the fact that the genome of durum and bread wheat do not show complete genetic collinearity, and that the races of STB that attack durum may be different from those attacking bread wheat.

On Chr 1A, a putative QTL was detected at ~68 cM (Figure 4). Two QTL for STB resistance in adult plants were already reported on this linkage group, one of which is *QTL1* (Goudemand et al., 2013) and the second is *Qstb.isa.fb-1A* (Risser et al., 2011). These QTL lay between 56 and 69 cM and could correspond to qSTB.1. On Chr 2B, a putative QTL was identified at around 85.8 cM (Figure 4). Several QTL and meta-QTL for STB resistance were previously reported in the proximity of our putative QTL qSTB.2 (Goudemand et al., 2013; Radecka-Janusik and Czembor, 2014). This locus is likely co-localized with the resistance gene *Stb9*, a major source of STB resistance (Chartrain et al., 2009). The combined measures of SPC identify qSTB.4. Its genetic position is matching that of the resistance locus *Stb7* (McCartney et al., 2003), reported on Chr 4AL using microsatellite loci. In addition to previously reported loci, we identified novel putative QTL for STB resistance. This was expected, since the broad and novel allelic diversity presented by the Ethiopian landrace material (Mengistu et al., 2016). On Chr 3A several significant associations emerged around 71 cM (Table 2, Figure 4, Figure S5). The putative QTL qSTB.3 is found in a position close to a QTL previously described for resistance to different STB isolates in winter wheat (Eriksen et al., 2003; Tabib Ghaffary et al., 2011; Radecka-Janusik and Czembor, 2014). On the same chromosome, but 55 cM away, lies the resistance gene *Stb6*, the most common STB resistance locus in European bread wheat germplasm (Brading et al., 2002; Chartrain et al., 2005; Arraiano and Brown, 2006). Our signal is found on the long arm of Chr 3A, while *Stb6* and other QTL are located on the short arm of the same Chr. On Chr 4B, one suggestive association was detected at 4 cM for SDS at maturity (Figure S5). To the best of our knowledge, this putative QTL was never reported in the literature and may represent a resistance allele specific to Ethiopian material. qSTB.5, mapping at ~135 cM on Chr 5A, does not match the genetic position of *Stb17*, a Septoria resistance gene reported at 62 cM on the same Chr (Tabib Ghaffary et al., 2012). QTL for Septoria resistance are reported on the same linkage group on bread wheat, in the 7.6 cM centromeric region of Chr 5AL (Dreisigacker et al., 2015). On Chr 5B, a putative QTL was identified at 43 cM by an MTA with estimated map position deriving from measures of SDS at heading in 2012 (Table S4). The closest STB resistance gene reported in the literature on this linkage group is *Stb1*, located around 68 cM (Adhikari et al., 2004), 25 cM downstream our signal. Further studies are required to provide a deeper characterization of these loci and evaluate the extent of their overlap with STB resistance loci previously reported.

All Septoria disease severity (SDS) MTAs are identified from either combined or 2012 data. SDS from 2013 did not report significant associations. The situation is opposite with Septoria progress coefficient (SPC), that provides an MTA unique to 2013 (Table 2). Our experiment was conducted in Geregera, a location chosen because of historical records of STB outbursts. The growing seasons of 2012 and 2013 were characterized by a markedly different climate that may have influenced the severity of the disease. More intense rain and the persistent wet climate in the year 2012 across the entire growing season must have favored the natural infection by the pathogen. Heavy rain determines water splashes that favor pycnidia spreading in the field, resulting in a high level of STB in susceptible genotypes. This increased the discrimination power between susceptible landraces and landraces with various degree of resistance. This was not the case of the 2013 growing season, which witnessed a relatively low rainfall that generally reduced the infection level. Still, the disease was present, and allowed to identify qSTB.5 (Table 2). By comparing the trends of disease severity over 2012 and 2013, it is clear that whilst SDS generally decreases, SPC tends to increase for most of the varieties (Figure S1), providing support for the discrepancy in QTL mapping power of SDS and SPC across years. The different climatic conditions may have caused a later onset of the disease in 2013. This in turn may have resulted in a general reduction of disease severity, especially at heading date. However, many varieties show disease trends contrasting with the general variation of disease pressure in the field. Especially for SDS at maturity, several varieties show higher affection in 2013 than in 2012, even though SDS is generally lower in 2013. This contrasting trends suggest that different Septoria races may have attacked the field in the two subsequent seasons, determining a differential response from some of the lines. At the time of the experiment, there was very poor characterization of Ethiopian Septoria races, and differential lines were not available in local durum wheat. Further studies are needed to isolate the pathogen races, and to untangle their effect on Ethiopian traditional and modern varieties.

In some cases, putative QTL deriving from agronomic traits co-localized with those identified by STB resistance traits. The putative QTL identified by 1,000 grain weight on Chr 1A between 60 and 70 cM co-maps with qSTB.1 (Table 2). It is expected for SDS to affect seed filling by damaging the flag or second leaf (King et al., 1983): as a result, the weight of grains rather than the number of seeds is affected. This is consistent with the putative QTL identified for 1,000 grain weight but not for number of spikelets and number of seeds per spike. Conversely, a putative QTL mapped at 40 cM on Chr 5B was identified by 1,000 grain weight, seeds per spike, and by a previously unmapped MTA for SDS at heading measured in 2012. This putative QTL does not appear in combined measurements of disease traits nor in disease data collected in 2013 and requires further testing to be confirmed. A putative QTL for seeds per spike co-maps with qSTB.4, identified by SPC on Chr 4A. It is expected that the number seeds per spike produced is influenced by the progression rate of the disease. More studies decoupling the effect of disease traits from agronomic values are needed to further dissect these putative QTL. Grain yield *per se* was poorly correlated with disease severity (Figure 2) and did not provide any significant QTL, likely because of the limited environments tested in this study.

None of the putative QTL identified by plant height and phenology overlapped those identified by disease traits. The closest signal was detected by days to heading on Chr 2B, at ~5 cM upstream qSTB.2. The two putative QTL lay at a relatively large distance, greater than the LD decay threshold employed (Table S6), hence are different loci. Days to heading also reports a highly significant association at 3.9 cM on Chr 4B (Table S7) overlapping a suggestive signal detected by SDS at maturity (Figure S5), that, however, does not qualify as QTL since fails to reach the significance threshold. The fact that in our panel plant height and days to heading and maturity provide putative QTL not overlapping with those reported by disease traits suggests that there is not a common genetic base for disease susceptibility and plant structure and phenology in the tested material. According to literature, late genotypes are favored over early genotypes because of an advantageous combination with Septoria development cycle rather than because of an inherent resistance to the disease (Arama et al., 1999; Simón et al., 2004). It would be possible to completely control for the effect of plant structure and phenology considering them as covariates of the GWA scan. However, when we did so, no MTA would surpass the significance threshold. This is likely derived from limited statistical power deriving from the restricted phenotyping conducted in this study. Rather, we use SPC to control for plant structure over disease severity. Future works employing data collected over multiple years and locations will allow to incorporate measures of flowering and height as covariates in the association mapping model to further characterize the relation between disease susceptibility and plant structure in our panel.

The results here reported provide an overview of the resistance alleles for *S. tritici* found in Ethiopian durum wheat genotypes. Several putative QTL identified in our study had a similar map position to previously identified resistance genes and QTL, many of which described on bread wheat alone. Our GWA on tetraploid wheat confirmed the importance of STB resistance loci identified in hexaploid wheat. Perhaps more interestingly, we report loci for STB resistance previously undiscovered on common wheat germplasm, either bread or durum. Even though our GWA study relies on data collected in one location only, the large number of characterized genotypes and molecular markers allowed us to identify highly significant genomic loci involved in STB resistance. Our results confirm the relevance of Ethiopian durum wheat as a reservoir of useful alleles and calls for additional characterizations to be conducted on this material. The overlay of STB data collected in further locations and growing seasons will advance our understanding of fungal resistance in durum wheat. The forthcoming availability of the durum and bread wheat high-quality genome assemblies will speed up the functional characterization of such novel sources of resistance, allowing to characterize the predicted genes in LD with the reported MTA. The production and characterization of segregating populations derived from the Ethiopian landraces panel will allow the further dissection of the genetic bases of STB resistance, including testing non-additive effects such as epistatic interactions among QTL loci. We anticipate that we developed a multiparental mapping population from Ethiopian durum wheat landraces, and that this population is being also characterized for STB resistance. Altogether, the use of new genomic tools, new test environments, and new genetic materials will help breeders to identify and transfer complementary resistances from Ethiopian durum wheat landraces into wheat breeding material, and to contribute to STB resistance in fields worldwide.

## Author contributions

YK and BH conducted field experiments and phenotypic data analysis. BH scored disease severity. YK and DM contributed material and statistical analyses. MP and CF supervised the project, and with MD coordinated the research work. MD performed genetic data analysis and GWAS with YK, drafted the paper and produced graphical outputs. All authors approved the final version of the manuscript.

### Conflict of interest statement

The authors declare that the research was conducted in the absence of any commercial or financial relationships that could be construed as a potential conflict of interest.
