# Supplementary material for: Genome-Wide Association Study of Septoria tritici Blotch Resistance in Ethiopian Durum Wheat Landraces
Source: Front Plant Sci. 2017 Sep 14;8:1586. doi: 10.3389/fpls.2017.01586 (PMC5603693; doi:10.3389/fpls.2017.01586)
Supplement: Supplementary file 10 [file Figure1.PPTX]

## Slide 1
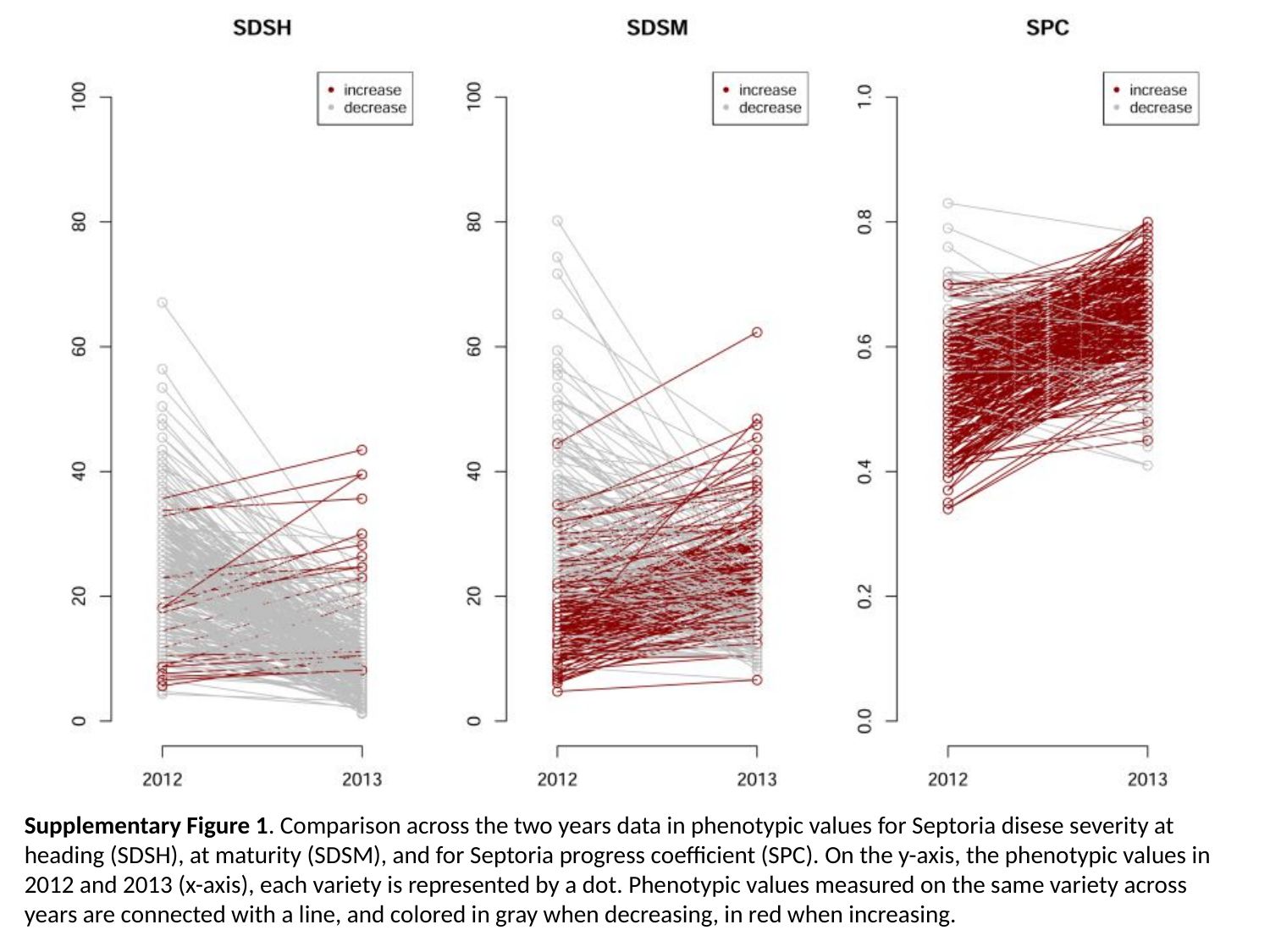

Supplementary Figure 1. Comparison across the two years data in phenotypic values for Septoria disese severity at heading (SDSH), at maturity (SDSM), and for Septoria progress coefficient (SPC). On the y-axis, the phenotypic values in 2012 and 2013 (x-axis), each variety is represented by a dot. Phenotypic values measured on the same variety across years are connected with a line, and colored in gray when decreasing, in red when increasing.

## Slide 2
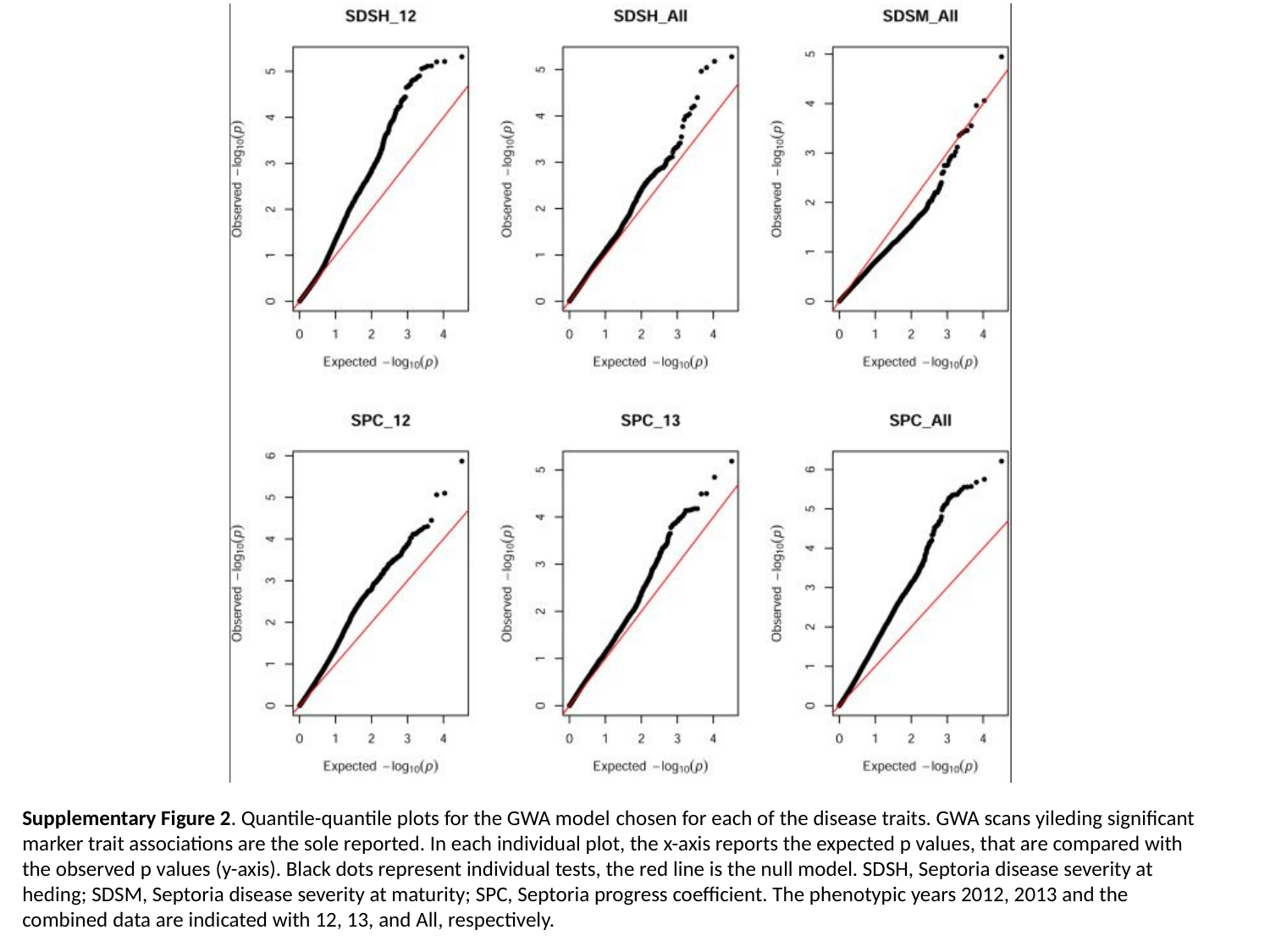

Supplementary Figure 2. Quantile-quantile plots for the GWA model chosen for each of the disease traits. GWA scans yileding significant marker trait associations are the sole reported. In each individual plot, the x-axis reports the expected p values, that are compared with the observed p values (y-axis). Black dots represent individual tests, the red line is the null model. SDSH, Septoria disease severity at heding; SDSM, Septoria disease severity at maturity; SPC, Septoria progress coefficient. The phenotypic years 2012, 2013 and the combined data are indicated with 12, 13, and All, respectively.

## Slide 3
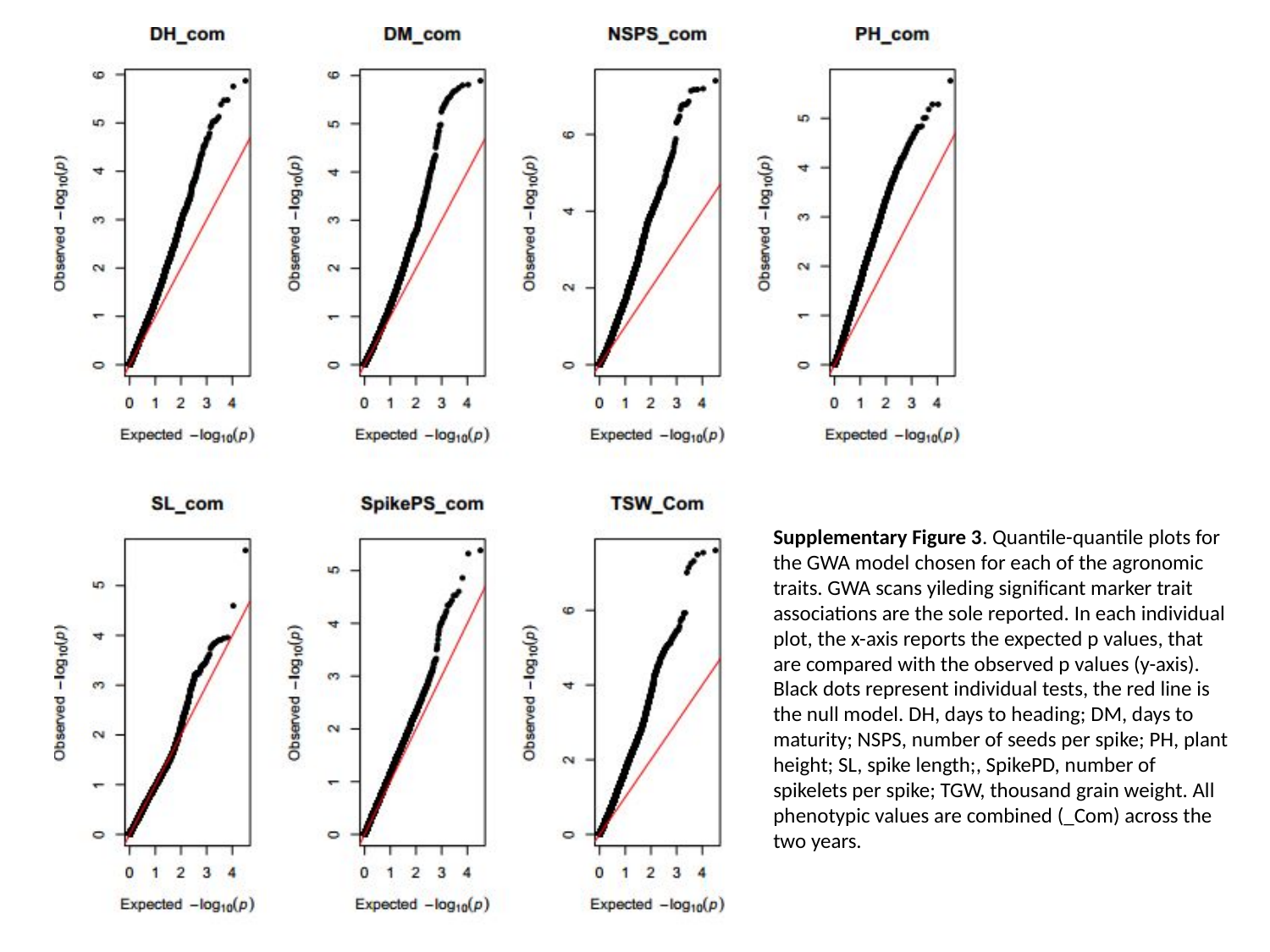

Supplementary Figure 3. Quantile-quantile plots for the GWA model chosen for each of the agronomic traits. GWA scans yileding significant marker trait associations are the sole reported. In each individual plot, the x-axis reports the expected p values, that are compared with the observed p values (y-axis). Black dots represent individual tests, the red line is the null model. DH, days to heading; DM, days to maturity; NSPS, number of seeds per spike; PH, plant height; SL, spike length;, SpikePD, number of spikelets per spike; TGW, thousand grain weight. All phenotypic values are combined (_Com) across the two years.

## Slide 4
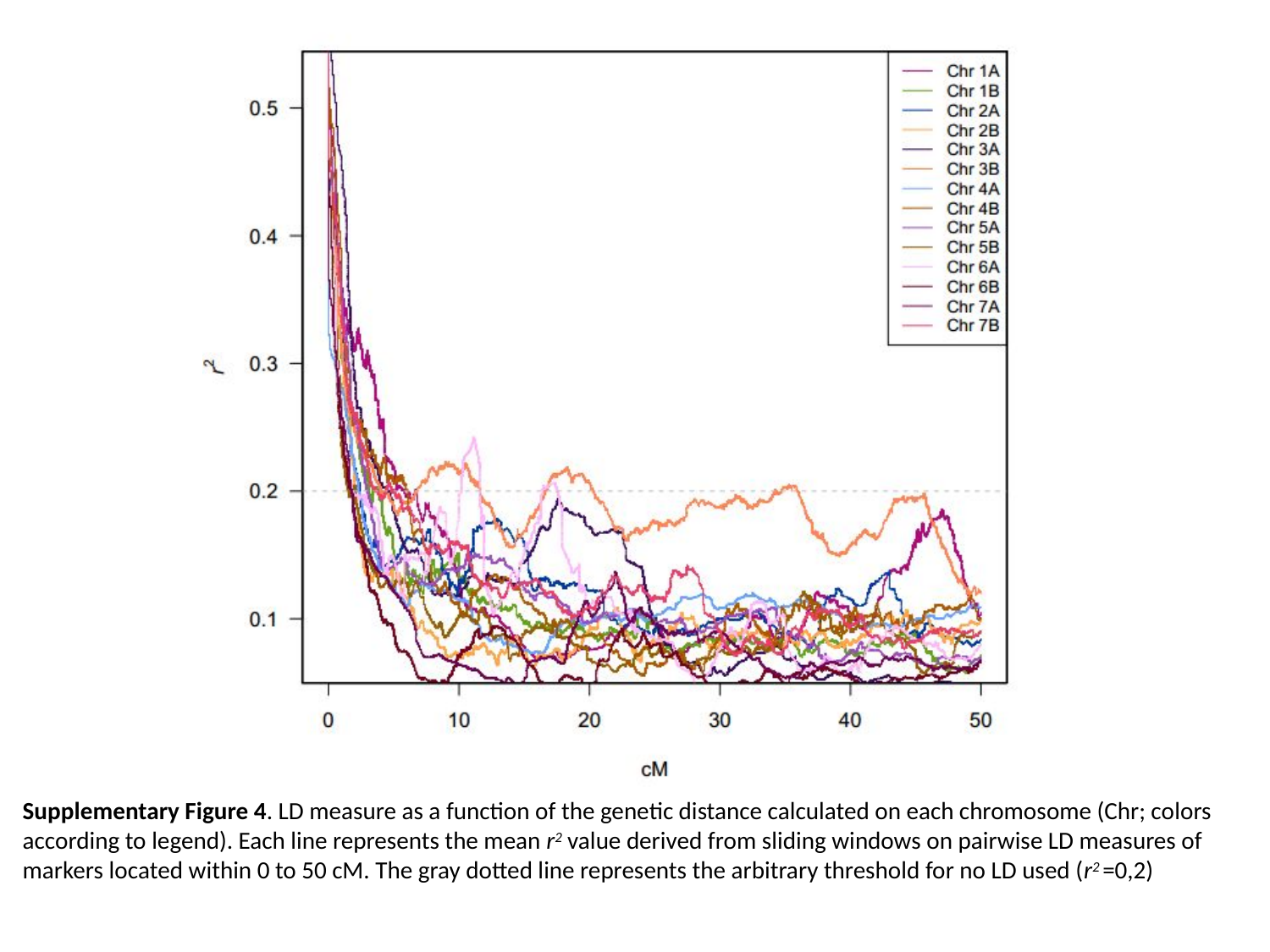

Supplementary Figure 4. LD measure as a function of the genetic distance calculated on each chromosome (Chr; colors according to legend). Each line represents the mean r2 value derived from sliding windows on pairwise LD measures of markers located within 0 to 50 cM. The gray dotted line represents the arbitrary threshold for no LD used (r2 =0,2)

## Slide 5
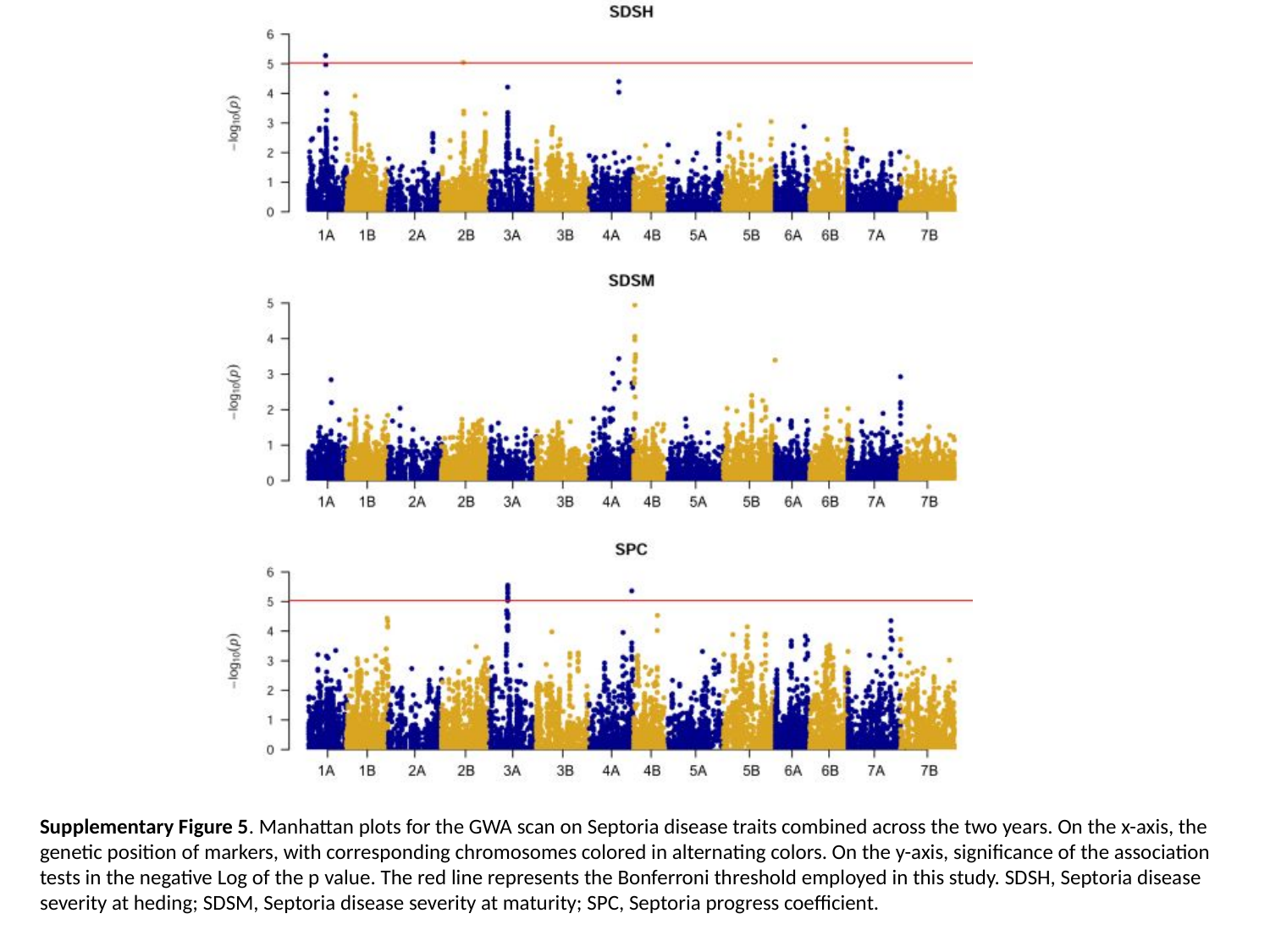

Supplementary Figure 5. Manhattan plots for the GWA scan on Septoria disease traits combined across the two years. On the x-axis, the genetic position of markers, with corresponding chromosomes colored in alternating colors. On the y-axis, significance of the association tests in the negative Log of the p value. The red line represents the Bonferroni threshold employed in this study. SDSH, Septoria disease severity at heding; SDSM, Septoria disease severity at maturity; SPC, Septoria progress coefficient.

## Slide 6
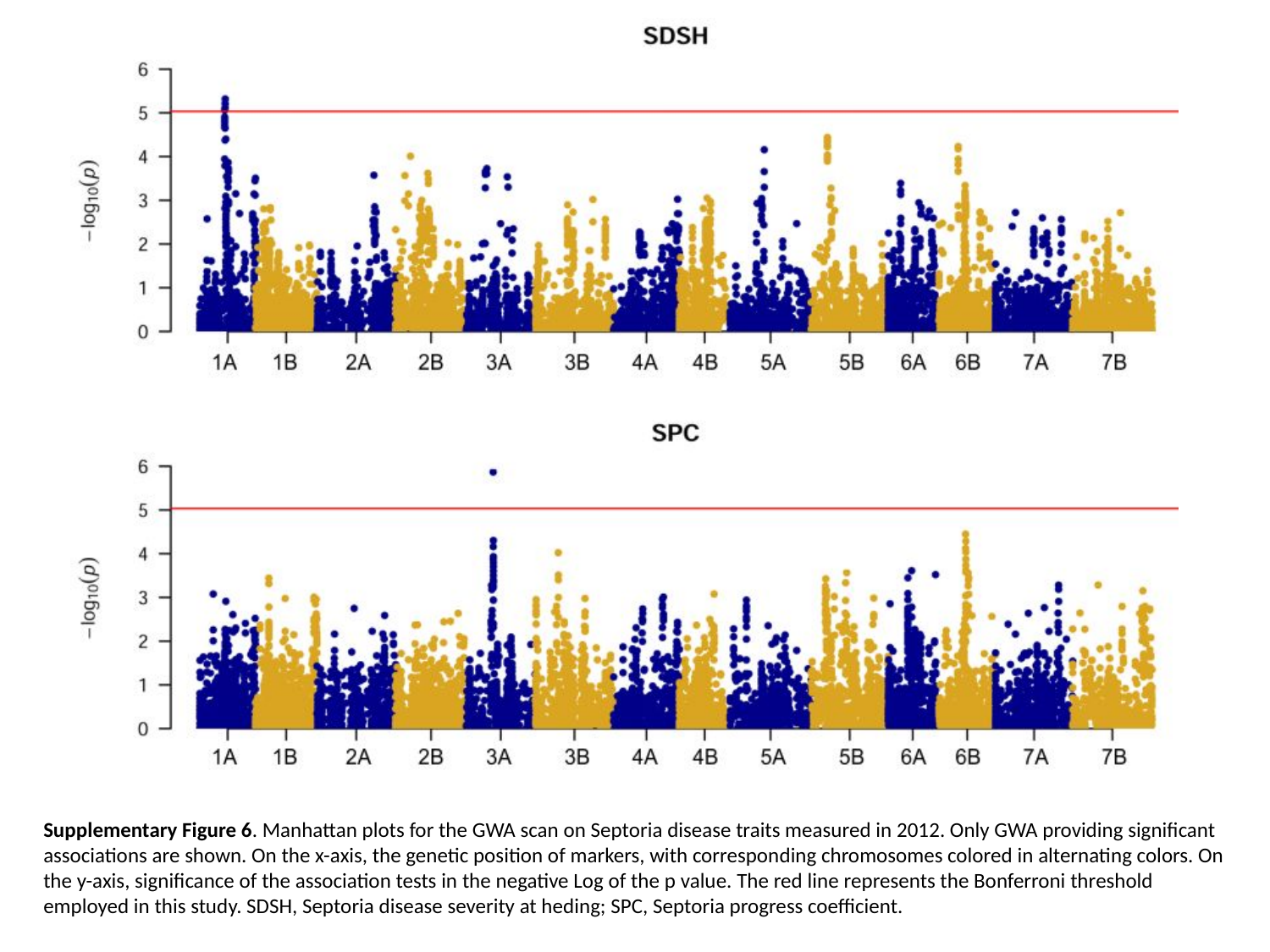

Supplementary Figure 6. Manhattan plots for the GWA scan on Septoria disease traits measured in 2012. Only GWA providing significant associations are shown. On the x-axis, the genetic position of markers, with corresponding chromosomes colored in alternating colors. On the y-axis, significance of the association tests in the negative Log of the p value. The red line represents the Bonferroni threshold employed in this study. SDSH, Septoria disease severity at heding; SPC, Septoria progress coefficient.

## Slide 7
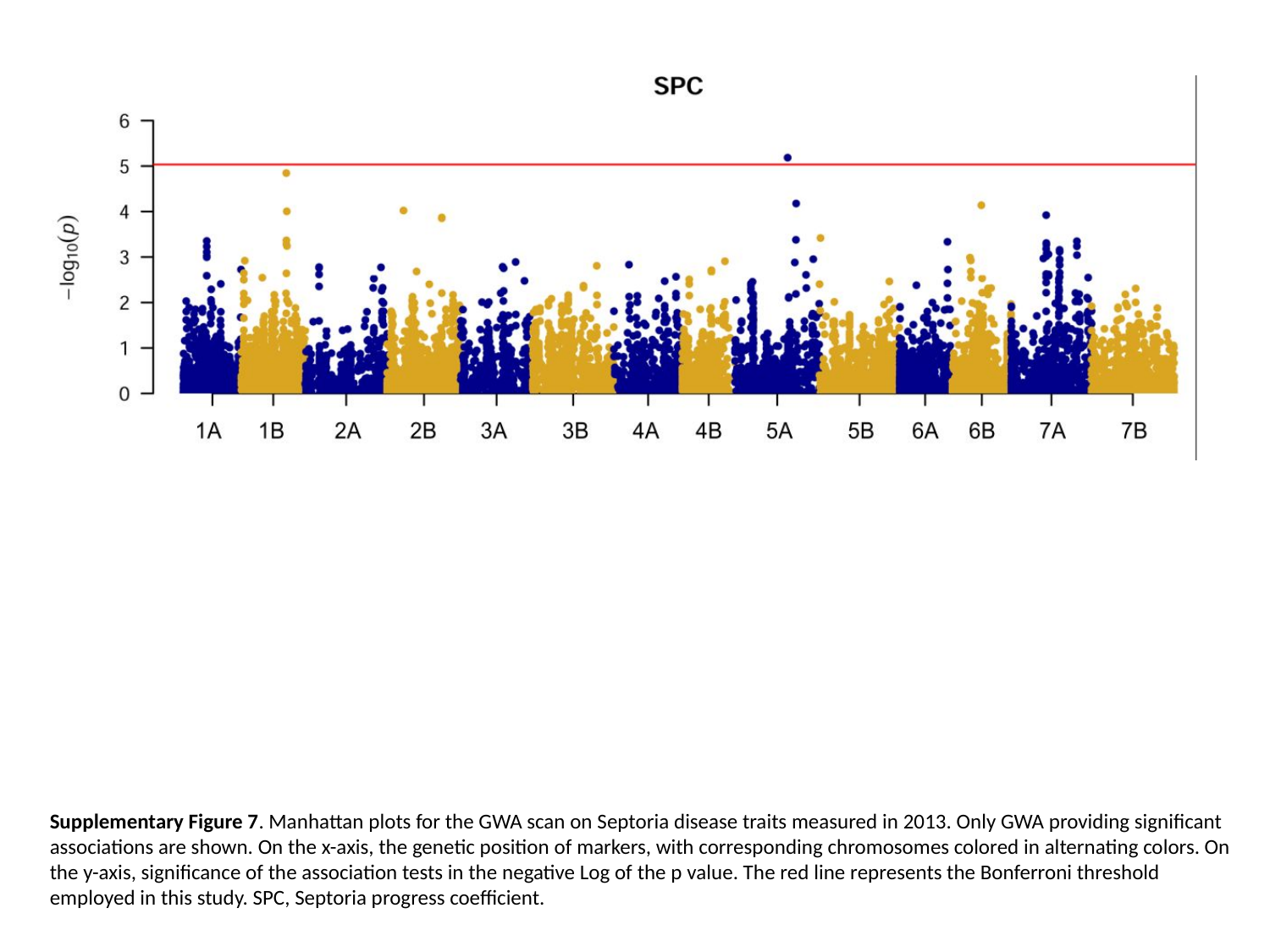

Supplementary Figure 7. Manhattan plots for the GWA scan on Septoria disease traits measured in 2013. Only GWA providing significant associations are shown. On the x-axis, the genetic position of markers, with corresponding chromosomes colored in alternating colors. On the y-axis, significance of the association tests in the negative Log of the p value. The red line represents the Bonferroni threshold employed in this study. SPC, Septoria progress coefficient.

## Slide 8
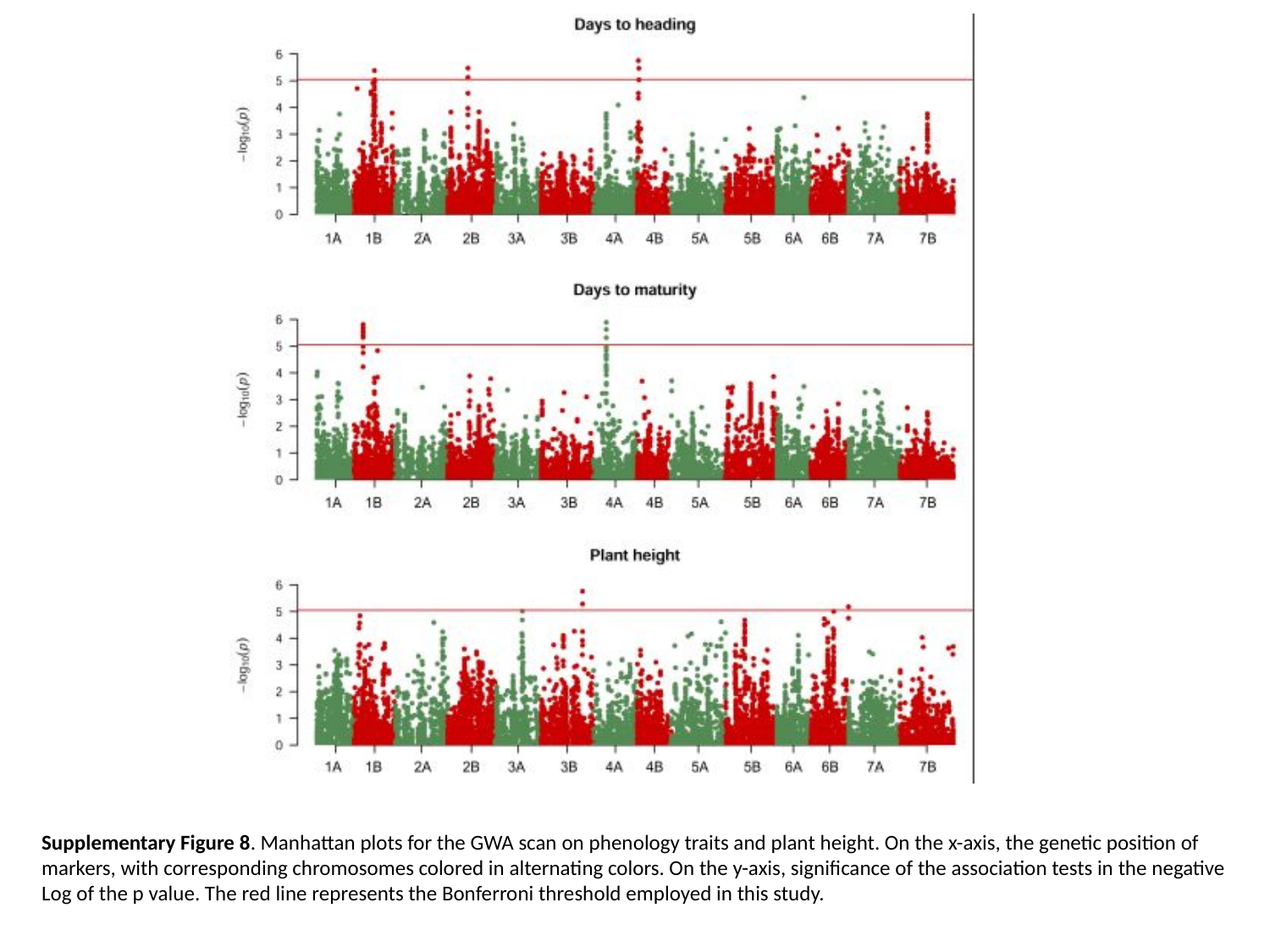

Supplementary Figure 8. Manhattan plots for the GWA scan on phenology traits and plant height. On the x-axis, the genetic position of markers, with corresponding chromosomes colored in alternating colors. On the y-axis, significance of the association tests in the negative Log of the p value. The red line represents the Bonferroni threshold employed in this study.

## Slide 9
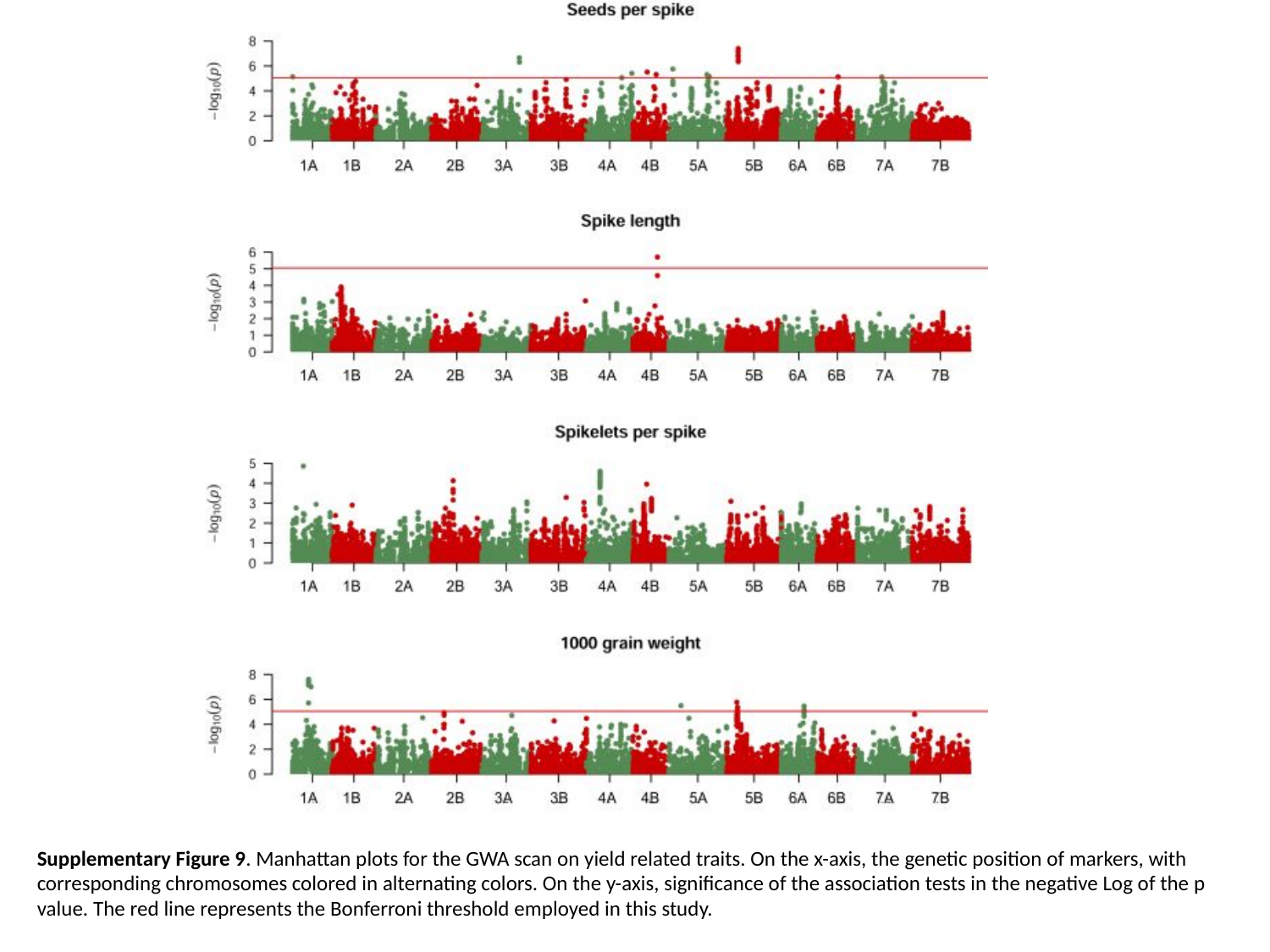

Supplementary Figure 9. Manhattan plots for the GWA scan on yield related traits. On the x-axis, the genetic position of markers, with corresponding chromosomes colored in alternating colors. On the y-axis, significance of the association tests in the negative Log of the p value. The red line represents the Bonferroni threshold employed in this study.
